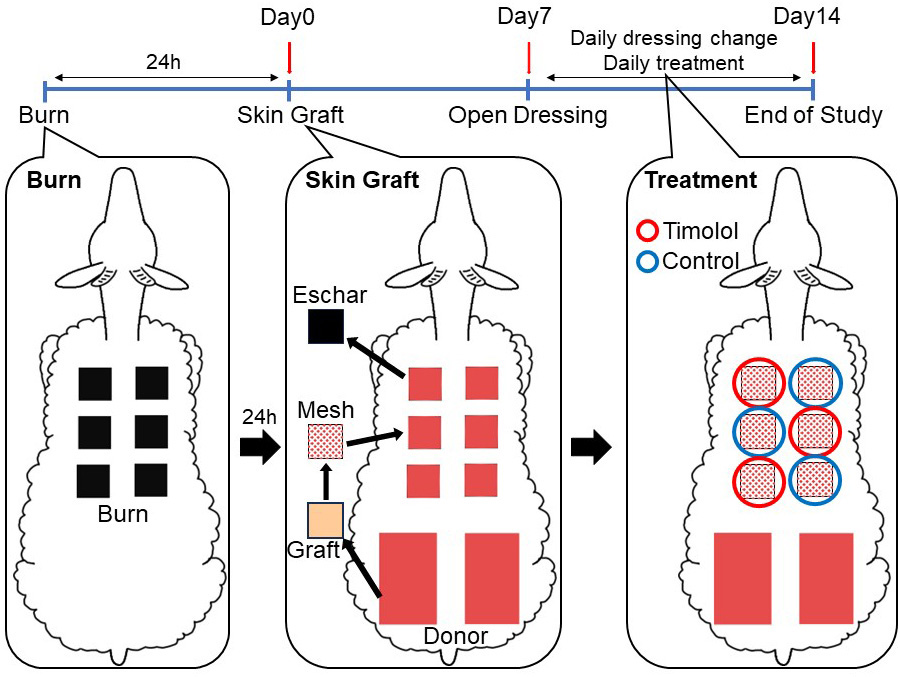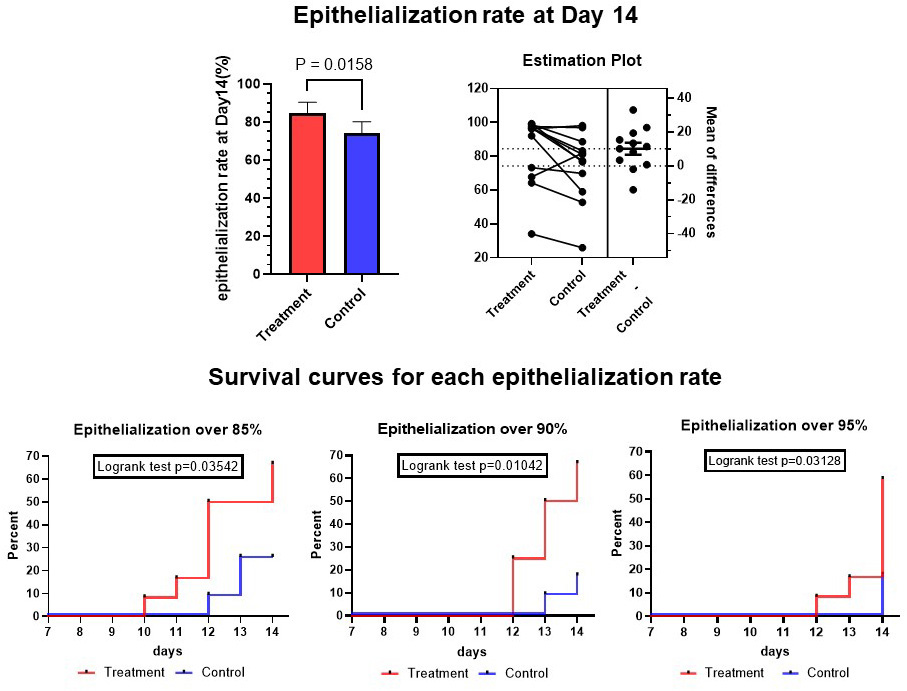# 556 Topical Application of Beta Blocker Promotes Healing of Mesh-grafted Third-Degree Burn Wounds in Sheep

**DOI:** 10.1093/jbcr/iraf019.185

**Published:** 2025-04-01

**Authors:** Kan Nakamoto, Tsend-Ayush Batsaikhan, Naiyou Liu, W Samuel Fagg, Ryuichiro Kakizaki, Thomas Heathman, Perenlei Enkhbaatar

**Affiliations:** University of Texas Medical Branch; University of Texas Medical Branch; University of Texas Medical Branch; University of Texas Medical Branch; University of Texas Medical Branch; University of Texas Medical Branch; University of Texas Medical Branch

## Abstract

**Introduction:**

Beta adrenergic receptor blockers are used for hypertension, tachycardia, and glaucoma. Recently, it has been shown to promote wound epithelialization. In this study, we tested the efficacy of the beta blocker timolol in an ovine model of grafted third degree burn wound healing which closely mimics a clinical scenario.

**Methods:**

Six third degree flame burn wounds were induced on the backs of the sheep. Twenty-four hours later, the eschars were excised and meshed skin was grafted onto the wound (Day 0). In the treatment group, timolol was applied topically to the wounds. Blood flow was measured with a blood perfusion imager. Cardiovascular hemodynamics and blood glucose levels were monitored. At Day14, the epithelialization rate was measured by planimetric assay and compared by paired t-test. Survival analysis was used to compare the days when epithelialization rates exceeded 85%, 90%, and 95% between the treatment and control groups. Differences in RNA abundance were measured by RT-qPCR to assess the potential contribution TGFβ-, epithelial-mesenchymal transition (EMT)-, and myofibroblast activation (MFA)-related processes.

**Results:**

The wound epithelialization rate at Day 14 in the treatment group (84.46±5.893 %) was significantly higher than in the control group (74.30±5.861 %) (P=0.0158). The days when the epithelialization rate exceeded 85, 90, and 95 % in the timolol group were significantly shorter than those in the control group (P=0.0354, P=0.0104, and P=0.0313, respectively). No significant difference was observed at any timepoints regarding wound blood flow or RNA abundance pertaining to TGFβ-, EMT-, or MFA-related pathways between the groups.

**Conclusions:**

The results show that the beta blocker timolol promotes epithelialization of mesh skin grafted third degree burn wounds. This is likely mediated by a mechanism independent of improved wound blood flow.

**Applicability of Research to Practice:**

Beta blockers could be used as therapeutic agents in the treatment of third degree burns in humans.

**Funding for the Study:**

Financial support: PE was supported by NIH GM097480.

Funding supports for WSF and NL were from the John L Hern University Chair in Transplant Surgery to WSF and the Mimmie and Hallie Smith Endowed Chair of Transplant Research to WSF.